# Role of the Prostate Imaging Quality PI-QUAL Score for Prostate Magnetic Resonance Image Quality in Pathological Upstaging After Radical Prostatectomy: A Multicentre European Study

**DOI:** 10.1016/j.euros.2022.11.013

**Published:** 2022-12-15

**Authors:** Olivier Windisch, Daniel Benamran, Charles Dariane, Martina Martins Favre, Mehdi Djouhri, Maxime Chevalier, Bénédicte Guillaume, Marco Oderda, Marco Gatti, Riccardo Faletti, Valentin Colinet, Yolene Lefebvre, Sylvain Bodard, Romain Diamand, Gaelle Fiard

**Affiliations:** a Department of Urology, Grenoble Alpes University Hospital, Université Grenoble Alpes, Grenoble, France; b Geneva University Hospital, Geneva, Switzerland; c AP-HP, Hôpital Européen Georges Pompidou, Service d’Urologie, F-75015, Paris, France; d Université Paris Cité, F-75006, Paris, France; e Imagerive, ID Imagerie, Unilabs SA, Geneva, Switzerland; f Department of Radiology, Grenoble Alpes University Hospital, Université Grenoble Alpes, Grenoble, France; g Molinette Hospital, Città della Salute e della Scienza di Torino, University of Turin, Turin, Italy; h Institut Jules Bordet, Brussels, Belgium; i AP-HP, Hôpital Necker Enfants Malades, Service d’Imagerie Adulte, F-75015, Paris, France; j Sorbonne Université, CNRS UMR 7371, INSERM U 1146, Laboratoire d’Imagerie Biomédicale, LIB, F-75006, Paris, France

## Abstract

**Background:**

Increasing use of multiparametric magnetic resonance imaging (mpMRI) has come with heterogeneity in image quality. The Prostate Imaging Quality (PI-QUAL) score is under scrutiny to assess its usefulness in predicting clinical outcomes.

**Objective:**

To compare upstaging of localized disease on mpMRI (mrT2) to locally invasive disease in radical prostatectomy (RP) specimens (≥pT3a) in relation to PI-QUAL.

**Design, setting, and participants:**

Patients treated with RP between 2015 and 2020 who underwent 1.5–3-T mpMRI within 6 mo before surgery and had systematic and mpMRI-US targeted biopsies were included. mpMRI scans were retrospectively assigned a PI-QUAL score, and prospectively acquired Prostate Imaging-Recording and Data System (PI-RADS) scores (version 2.0 or 2.1) were used. PI-QUAL scores were categorized as nondiagnostic (PI-QUAL <3), sufficient (PI-QUAL 3), or optimal (PI-QUAL >3).

**Outcome measurements and statistical analysis:**

We assessed the relationship between the PI-QUAL score and upstaging using multivariate logistic regression. mpMRI, clinical, and pathological findings were compared using χ^2^ tests and analysis of variance.

**Results and limitations:**

We identified 351 patients, of whom 40 (11.4%) had PI-QUAL <3, 57 (16.3%) had PI-QUAL 3, and 254 (72.3%) had PI-QUAL >3 scores. The distribution of PI-QUAL <3 (0–33.6%; *p* < 0.001) and PI-QUAL >3 (37.3–100%; *p* < 0.001) scores varied widely among centers. PI-QUAL ≥3 in comparison to PI-QUAL <3 was associated with a lower rate of upstaging (19% vs 35%; *p* = 0.02), greater detection of mrT3a and mrT3b prostate cancer (17.0% vs 2.5%; *p* = 0.016), a higher rate of PI-RADS 5 lesions (47% vs 27.5%; *p* = 0.002), a higher number of suspicious lesion (PI-RADS ≥3: 34.7% vs 15%; *p* = 0.012), and higher detection rates for aggregated (50.7% vs 22.5%; *p* = 0.001) and late (21.2% vs 0%; *p* < 0.001) extraprostatic extension. On multivariate analysis, PI-QUAL<3 was associated with more frequent upstaging in the RP specimen (odds ratio 3.4; *p* = 0.01).

**Conclusions:**

In comparison to PI-QUAL ≥3, PI-QUAL <3 was significantly associated with a higher rate of upstaging from organ-confined disease on mpMRI to locally advanced disease on pathology, lower detection rates for PI-RADS 5 lesions and extraprostatic extension, and a lower number of suspicious lesions.

**Patient summary:**

Poor image quality for magnetic resonance imaging (MRI) scans of the prostate is associated with underestimation of the stage of prostate cancer.

## Take Home Message

PI-QUAL (Prostate Imaging Quality) is a new score used to standardize the assessment of multiparametric magnetic resonance imaging (mpMRI). We provide further evidence that PI-QUAL has clinical implications. mpMRI of insufficient quality, defined as PI-QUAL <3, results in a higher rate of pathological upstaging.

## 1 Introduction

Multiparametric magnetic resonance imaging (mpMRI) is recommended by the European Association of Urology for patients at risk of prostate cancer (PCa), as it may reduce the number of unnecessary biopsies while detecting more clinically significant prostate cancer (csPCa) and less clinically insignificant PCa [1], [2]. The Prostate Imaging-Reporting and Data System (PI-RADS) was first described in 2012 to provide minimum technical standards for better imaging quality and to standardize imaging interpretation, and has been regularly updated since then, with the latest revision (version 2.1) published in 2019 [3]. Even with these recommendations, the increase in mpMRI use has come with heterogeneity in imaging quality and variability in interpretation among centers [4]. Since image acquisition and interpretation impact the whole diagnostic process, concerns have been raised that PI-RADS recommendations may not be sufficient to ensure that optimal images are obtained [5]. This led to the creation of the first standardized scoring system for image quality, Prostate Imaging Quality (PI-QUAL), which is used to assess the quality of mpMRI sequences on 5-point Likert score and assign a corresponding grade [6]. PI-QUAL 1–2 represents nondiagnostic quality (ie, it is not possible to rule in or to rule out all significant lesions), PI-QUAL 3 represents sufficient quality (ie, it is possible to rule in all significant lesions, but it is not possible to rule out all significant lesions), and PI-QUAL 4–5 represents optimal quality (ie, it is possible to rule in or rule out all significant lesions) [6].

In addition, it has been shown that mpMRI has low sensitivity but high specificity for predicting locally advanced disease in radical prostatectomy specimens [7]. There is no consensus regarding radiological findings suggestive of locally advanced disease; several signs and degrees of extraprostatic extension (EPE) have been described and different scores were reported to predict the risk of locally advanced disease on the basis of mpMRI findings [8]. No trial had investigated whether EPE detection is associated with mpMRI quality until a recent study suggested higher diagnostic performance for EPE detection and exclusion of locally advanced cancer with increasing PI-QUAL score [9].

The aim of our multicenter European study was to investigate whether imaging quality, according to the PI-QUAL score, is associated with upstaging between mpMRI staging and pathological staging.

## 2 Patients and methods

### 2.1 Study design, participating centers, and inclusion period

We conducted a multicenter retrospective analysis of patients undergoing radical prostatectomy who had an mpMRI examination between January 1, 2015 and December 31, 2020. Five European tertiary referral centers were included in the study after obtaining approval from their institutional review boards.

### 2.2 Inclusion and exclusion criteria

The inclusion criteria were as follows: patients treated with radical prostatectomy (open, laparoscopic, or robotic) during the study period; 1.5- or 3-T mpMRI within 6 mo before surgery (with or without an endorectal coil); mpMRI-ultrasound–targeted (≥2 targeted biopsies per lesion) and systematic biopsies; and histological analysis of the prostatectomy specimen according to International Society of Urological Pathology (ISUP) 2014/World Health Organization 2016 guidelines. Patients had to meet all the criteria to be included.

The exclusion criteria were previous hormone therapy, radiotherapy, or transurethral/open prostate resection before the radical prostatectomy surgery, as these could affect radiological interpretation.

### 2.3 mpMRI analysis

In each center, an expert radiologist (≥1000 mpMRI reads overall and ≥200 mpMRI reads/yr) or a radiologist supervised by an expert radiologist (Supplementary Table 1) retrospectively analyzed previously acquired images (acquired in either the tertiary referral hospital or in an external imaging center) according to PI-RADS version 2.0 or 2.1 guidelines and attributed a PI-QUAL score [6] and defined an mrT stage according to the European Society of Urogenital Radiology/EAU Section of Urologic Imaging consensus for expert radiologists [10]. The PI-QUAL score was divided into three clinical categories: nondiagnostic (PI-QUAL 1–2), sufficient (PI-QUAL 3), or optimal quality (PI-QUAL 4–5). The radiologists were blinded to the pathological data. The index lesion was defined as the lesion with the highest PI-RADS score or the lesion with the greatest diameter in the case of multiple lesions with the same score. The lesion location (base, mid, or apex) was identified on an axial scan and anatomic sequence (T2-wighted imaging). EPE was assessed using the Pesapane classification [11]. Early EPE was defined as any of the following: broad tumor contact with the capsule, smooth capsular bulging, capsular signal intensity disruption, or a margin that is not sharp. Late EPE was defined as any of the following: irregular contour, periprostatic fat infiltration, obliteration of the rectoprostatic angle, or tumor in periprostatic fat [11]. Patients presenting with late EPE were staged as mrT3a, whereas seminal vesicle invasion was defined as mrT3b. Aggregated EPE refers to the presence of either early or late signs of EPE.

### 2.4 Demographic and pathological data

Data regarding patient demographics, preoperative prostate-specific antigen level, clinical staging, biopsy, and histopathology for prostatectomy specimens were collected from computerized institutional medical records. The processing and reporting of the biopsies were performed according to the 2014 ISUP recommendations [12]. The presence and location of pathological EPE were included in the histopathology report. After extracting data from each center, the data were coded and anonymously transmitted for aggregated analysis.

### 2.5 Primary and secondary outcomes

The primary outcome of the study was the rate of upstaging among the different PI-QUAL groups, defined as patients presenting with an mpMRI-confined lesion (mrT2) for which the pathology report indicated a locally invasive lesion (≥pT3a).

Secondary outcomes were the distribution of PI-QUAL scores among centers; the rate of inconclusive imaging (PI-QUAL ≤3); and the relationship between the PI-QUAL score (nondiagnostic, sufficient, or optimal quality) and the PI-RADS score, EPE rate, number of suspicious lesions (defined as PI-RADS ≥3), upstaging of targeted biopsies compared to systematic biopsies, and the percentage of biopsies (systematic and targeted) with csPCa (ISUP grade group ≥2).

### 2.6 Statistical analysis

The Pearson χ^2^ test was used to compare categorical variables or Fisher’s exact test when appropriate; results are presented as the frequency and proportion. Results for continuous variables are presented as the median and interquartile range unless stated otherwise. Analysis of variance was used for comparison of continuous variables for more than two groups. The level of significance was set to *p* < 0.05 in two-tailed tests.

Pathological staging (pT3a or pT3b) was used as the reference to define locally advanced PCa and to evaluate sensitivity, specificity, positive and negative predictive values, and area under the receiver operating characteristic curve (AUC) for early, late, and aggregated EPE.

A univariate logistic regression model was used to test relevant clinical variables, including the PI-QUAL category, for upstaging from mpMRI (mrT2) to locally advanced disease on pathology (≥pT3a); relevant variables were further tested using multivariable logistic regression. Statistically significant variables (*p* < 0.05) were included in the final multivariable logistic model after testing of the application conditions.

## 3 Results

### 3.1 Patient demographics and PI-QUAL distribution

**Fig. 1:**
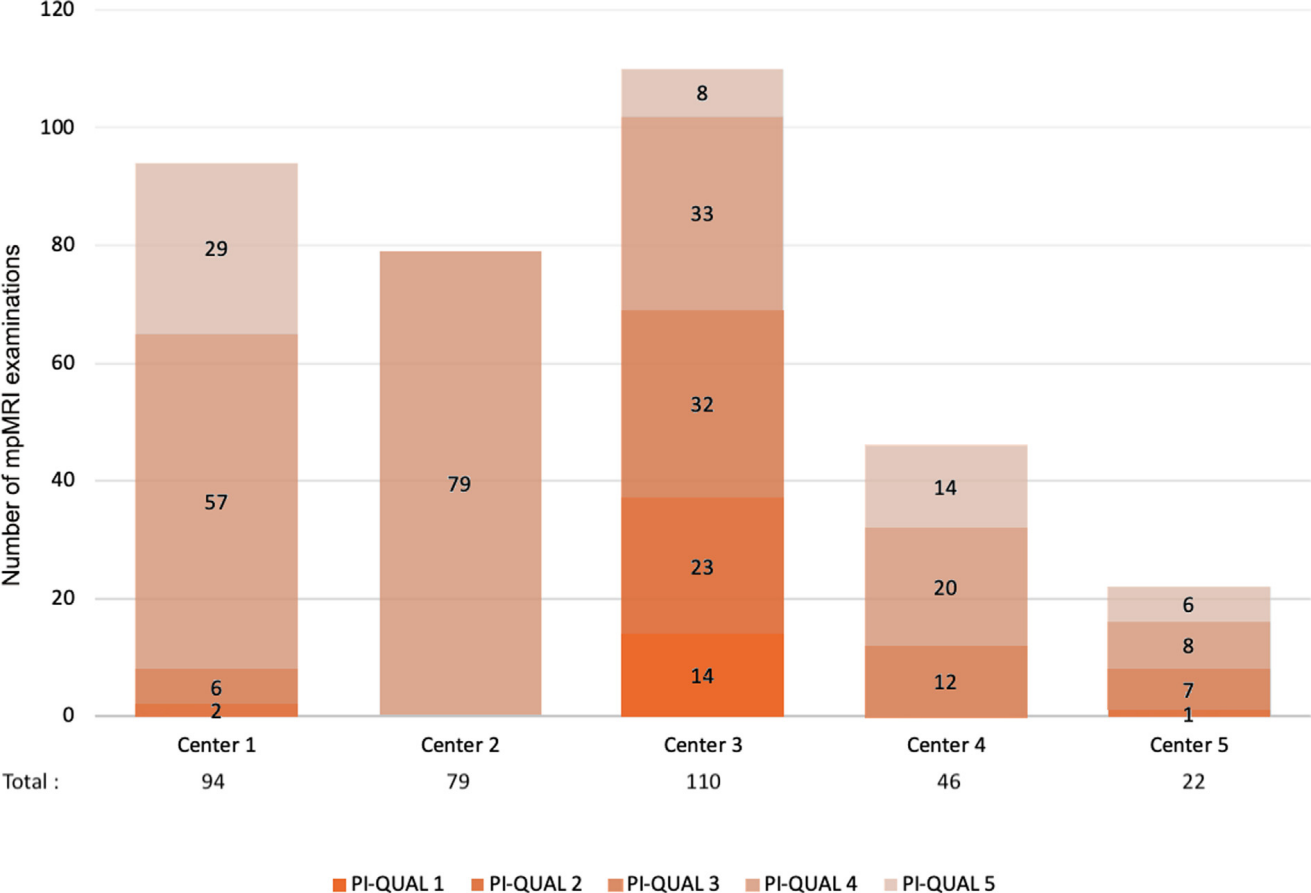
Prostate Imaging Quality (PI-QUAL) score distribution among centers. mpMRI = multiparametric magnetic resonance imaging.

**Fig. 2:**
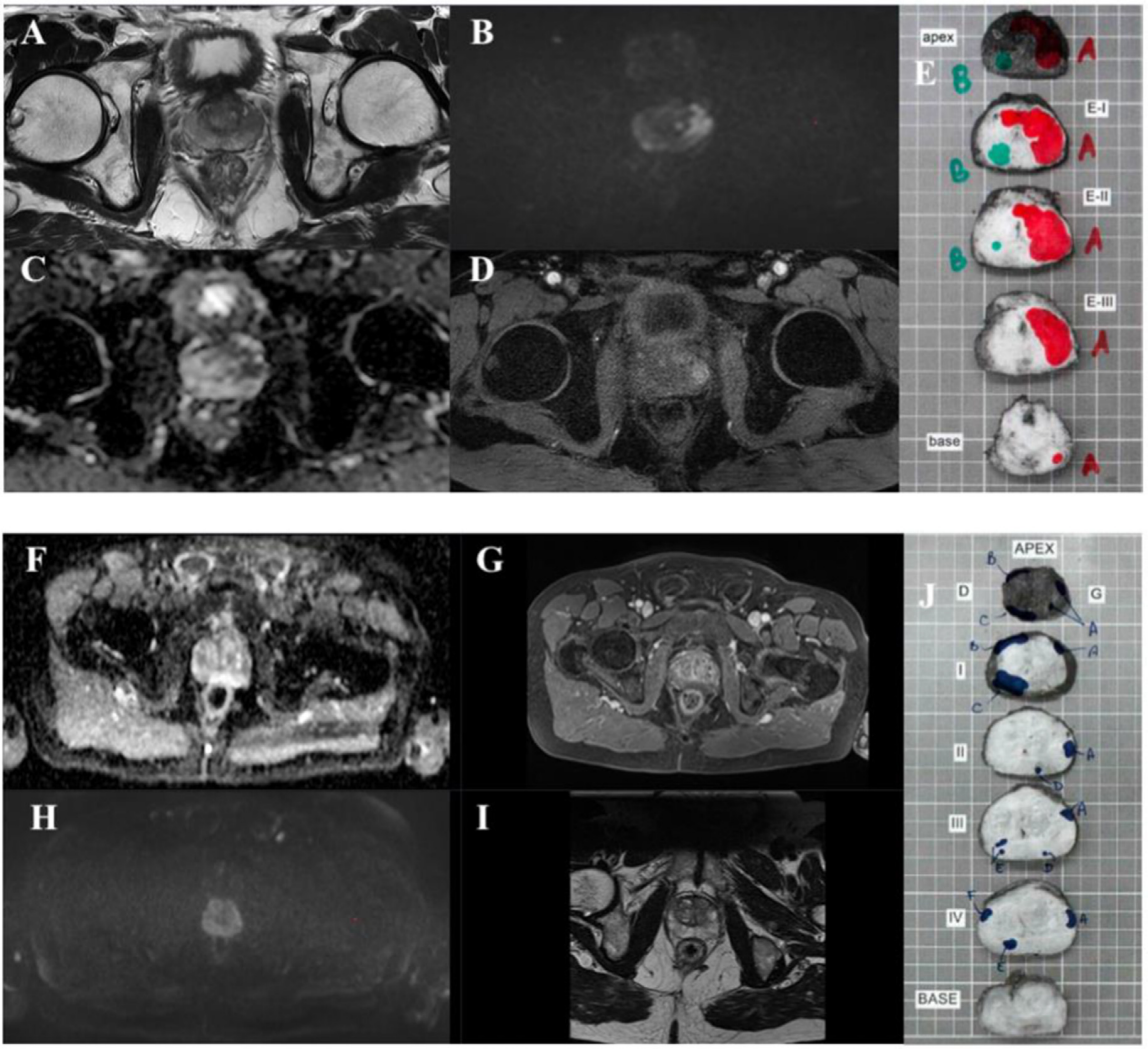
Visual comparison of nondiagnostic versus optimal quality according to the PI-QUAL scoring system. (A–E) PI-QUAL 5. (A) T2WI sequence, axial plane of adequate quality showing an 18-mm nodule of low signal intensity located in the left lateral middle part of the prostate. (B) DWI sequence of adequate quality with a high *b* value (1500 s/mm^2^) exhibiting restriction of the diffusion as shown by high signal intensity. (C) ADC sequence with the same slice thickness as for the T2 axial view and FOV, showing intense restriction of the diffusion. (D) DCE sequence of adequate quality with adequate fat suppression and acquisition for 4 min showing a high signal intensity during the early phase of the acquisition. (E) Axial cuts of a radical prostatectomy specimen from the apex to the base (top to bottom); two distinct foci were identified, with tumor shown in red and green. All sequences are concordant for a Prostate Imaging-Recording and Data System score of 5, with a high suspicious of malignancy. All sequences are adequate and have optimal diagnostic quality; the final PI-QUAL score is 5. (F–J) PI-QUAL 2. (F) ADC interpretation is limited because of kinetic artifacts and air in the rectum. (G) DCE sequence of inadequate quality because the definition of the prostatic capsule is unclear and the temporal resolution was too long (13 s). (H) DWI of inadequate quality because the FOV is too large (34 cm × 26 cm) and the high *b* value is too low (800 s/mm^2^). (I) T2WI axial view of adequate quality with adequate FOV, in-plane resolution, slice thickness, and correct *z*-axis position. (J) Axial cuts of a prostatectomy specimen from the apex to the base (top to bottom); six distinct foci were identified, with tumor shown in blue. As only the T2WI sequence has acceptable diagnostic quality, the final PI-QUAL score is 2. ADC = apparent diffusion coefficient; DCE = dynamic contrast-enhanced; FOV = field of view; PI-QUAL = Prostate Imaging Quality; T2WI = T2-weighted imaging.

A total of 351 patients were included in the study. Preoperative demographic characteristics are listed in Table 1. mpMRI of at least sufficient quality (PI-QUAL ≥3) was available for 311 patients (88.6%), while 40 (11.4%) had mpMRI scans of nondiagnostic quality (PI-QUAL <3). Wide heterogeneity in the distribution of PI-QUAL scores was observed among the different participating centers; the incidence of nondiagnostic quality ranged from 0% to 33.6% among centers (*p* < 0.001), while the incidence of optimal quality varied between 37.3% and 100% (*p* < 0.001). The contributions of the individual centers and the PI-QUAL distribution by center are presented in Figure 1. Figure 2 shows examples of imaging quality according to the PI-QUAL classification.

**Table 1:** Patient demographics

Parameter	Result
Patients (*n*)	351
Median age, yr (interquartile range)	66.3 (61.1–70.4)
Median prebiopsy PSA, ng/ml (interquartile range)	7 (5.3–10)
Median prostate volume, ml (interquartile range)	39.6 (30.9–52.5)
Median PSA density, ng/ml/ml (interquartile range)	0.16 (0.11–0.29)
Clinical T stage, *n* (%)	
cT1	201 (57.6)
cT2	134 (38.4)
cT3	14 (4.0)
Data missing	2

PSA = prostate-specific antigen.

### 3.2 mpMRI findings

Globally, comparison of mpMRI staging (Table 2) to pathological staging (Table 3) revealed that mpMRI underestimated the disease in 73 cases (20.8%). Pathological upstaging was less frequent for PI-QUAL 3 and PI-QUAL >3 than for PI-QUAL <3 cases (17.5% vs 19.3% vs 35%; *p* = 0.06; Table 4) but only reached statistical significance for PI-QUAL ≥3 versus PI-QUAL <3 (19% vs 35%; *p* = 0.02). While the distribution of pathological stages was similar between all PI-QUAL groups, significantly higher rates of mrT3a and mrT3b were observed for PI-QUAL >3 and PI-QUAL 3 than for PI-QUAL <3 cases (26.4% vs 17.6% vs 2.5%; *p* = 0.002). A higher frequency of multiple suspicious lesions was detected in the PI-QUAL >3 and PI-QUAL 3 groups in comparison to PI-QUAL <3 (35.0% vs 33.3% vs 15%; *p* = 0.04). Detection of aggregated EPE (54.0% vs. 40.3% vs 22.5%; *p* < 0.001) and late EPE (24.0% vs. 15.8% vs. 0%; *p* = 0.001) was significantly more frequent in the PI-QUAL >3 and PI-QUAL 3 groups than for PI-QUAL <3, whereas detection of early EPE did not differ significantly (Table 2). Differences in PI-RADS distribution were observed, with a higher proportion of PI-RADS 5 scores in the PI-QUAL >3 and PI-QUAL 3 groups than for PI-QUAL <3 (48% vs 42.1% vs 27.5%; *p* = 0.048).

**Table 2:** mpMRI findings in relation to PI-QUAL score

	PI-QUAL 1–2 (*n* = 40)	PI-QUAL 3 (*n* = 57)	PI-QUAL 4–5 (*n* = 254)	*p* value
**PIRADS v2.1 score, *n* (%)**				0.011
PI-RADS <3	2 (5)	0	1 (0.4)	
PI-RADS 3	5 (12.5)	3 (5.3)	13 (5.1)	
PI-RADS 4	22 (55)	30 (52.6)	118 (46.5)	
PI-RADS 5	11 (27.5)	24 (42.1)	122 (48.0)	
**mpMRI staging**				0.015
T stage, *n* (%)				
mrT2	39 (97.5)	47 (82.5)	187 (73.6)	
mrT3a	1 (2.5)	8 (14.0)	55 (21.7)	
mrT3b	0 (0)	2 (3.5)	12 (4.7)	
N stage, *n* (%)				0.881
N0	38 (95.0)	55 (96.5)	243 (95.7)	
N1	2 (5.0)	2 (3.5)	11 (4.3)	
EPE, *n* (%)				
No EPE	31 (77.5)	34 (59.6)	118 (46.5)	0.001
Early EPE	9 (22.5)	14 (24.6)	75 (29.5)	0.54
Late EPE	0	9 (15.8)	61 (24.0)	0.001
Median ROI size, mm (IQR)	12.5 (10.5–17.5)	13.0 (10–16)	13 (10–17)	0.827
Targets, *n* (%)				0.04
Unique	34 (85)	38 (66.7)	165 (65.0)	
Multiple	6 (15)	19 (33.3)	89 (35.0)	

EPE = extraprostatic extension; IQR = interquartile range; mpMRI = multiparametric magnetic resonance imaging; PI-QUAL = Prostate Imaging Quality score (nondiagnostic, PI-QUAL 1–2; sufficient, PI-QUAL 3; optimal, PI-QUAL 4–5); PI-RADS = Prostate Imaging-Reporting and Data System; ROI = region of interest.

**Table 3:** Histopathology for prostatectomy specimens by PI-QUAL score for multiparametric magnetic resonance imaging

Parameter	Patients, *n* (%)			*p* value
	PI-QUAL 1–2 (*n* = 40)	PI-QUAL 3 (*n* = 57)	PI-QUAL 4–5 (*n* = 254)	
T stage				
pT2	26 (65.0)	39 (68.4)	163 (64.1)	0.911
pT3a	10 (25.0)	15 (26.3)	71 (28.0)	
pT3b	4 (10.0)	3 (5.3)	20 (7.9)	
N stage				0.01
pN0	15 (37.5)	32 (56.1)	173 (68.1)	
pN1	3 (7.5)	1 (1.8)	14 (5.5)	
pNX	22 (55.0)	24 (42.1)	67 (26.4)	
ISUP grade group				0.629
Grade group 1	7 (17.5)	7 (12.3)	29 (11.4)	
Grade group 2	15 (37.5)	30 (52.6)	124 (48.8)	
Grade group 3	11 (27.5)	13 (22.8)	74 (29.1)	
Grade group 4	6 (15.0)	5 (8.8)	17 (6.7)	
Grade group 5	1 (2.5)	2 (3.5)	10 (4.0)	

ISUP = International Society of Urological Pathology; PI-QUAL = Prostate Imaging Quality score (nondiagnostic, PI-QUAL 1–2; sufficient, PI-QUAL 3; optimal, PI-QUAL 4–5).

**Table 4:** Agreement between multiparametric magnetic resonance imaging and pathological staging

Outcome	Patients, *n* (%)			*p* value
	PI-QUAL 1–2 (*n* = 40)	PI-QUAL 3 (*n* = 57)	PI-QUAL 4–5 (*n* = 254)	
Agreement	25 (62.5)	45 (79.0)	180 (70.9)	0.206
Pathological upstaging a	14 (35)	10 (17.5)	49 (19.3)	0.06
Pathological downstaging b	1 (2.5)	2 (3.5)	25 (9.8)	0.111

PI-QUAL = Prostate Imaging Quality score (nondiagnostic, PI-QUAL 1–2; sufficient, PI-QUAL 3; optimal, PI-QUAL 4–5).

a Pathological upstaging is locally advanced disease on pathology for the prostatectomy specimen (pT3a or pT3b) for cancer clinically diagnosed as mrT2 stage.

b Pathological downstaging is pT2 stage on pathology for the prostatectomy specimen for cancer clinically diagnosed as locally advanced disease on multiparametric magnetic resonance imaging (mrT3a or mrT3b).

While the differences between PI-QUAL <3 and PI-QUAL ≥3 were significant for all the parameters investigated, this was not the case when comparing sufficient quality (PI-QUAL 3) to optimal quality (PI-QUAL >3). There were no significant differences between PI-QUAL 3 and PI-QUAL >3 for pathological upstaging (17.5% vs 19.3%; *p* = 0.761) or the rates of mrT3a and mrT3b (17.5% vs 26.4%; *p* = 0.163), PI-RADS 5 (42% vs 48%; *p* = 0.418), late EPE (15.8% vs 24%; *p* = 0.267), aggregated EPE (40.3% vs 54.0%, *p* = 0.064), and multiple suspicious lesions (33.3% vs 35%; *p* = 0.807).

### 3.3 Diagnostic accuracy of EPE

The sensitivity, specificity, and positive and negative predictive values of EPE (early, late, or aggregated) for prediction of locally advanced disease on pathology are shown in Supplementary Table 2. The presence of early EPE on adequate-quality mpMRI had low sensitivity (22–36%) and moderate to high specificity (66–85%) for prediction of locally advanced lesions. Late EPE, only visible on mpMRI scored as PI-QUAL 3 or PI-QUAL >3, had excellent specificity (87–95%) and low sensitivity (39–43%) for prediction of locally advanced disease on pathology.

### 3.4 Preoperative biopsies

The biopsy results did not differ significantly between the PI-QUAL groups (Supplementary Table 3).

### 3.5 Factors associated with upstaging on radical prostatectomy pathology

The regression results for upstaging to locally advanced disease at final pathology are shown in Table 5. Several preoperative variables were significantly associated with upstaging on pathology: PI-QUAL <3 (odds ratio 3.4; *p* = 0.01), early EPE, multiple suspicious lesions on mpMRI, base location for the lesion, and percentage of positive targeted and systematic biopsies. Univariate results are presented in Supplementary Table 4.

**Table 5:** Multivariable logistic regression model for preoperative variables associated with upstaging on pathology for patients with organ-confined disease on mpMRI

Parameter	OR (95% CI)	*p* value
**mpMRI criteria**		
Imaging quality (vs PI-QUAL 3)		0.012
PI-QUAL 1–2	3.4 (1.2–9.3)	0.020
PI-QUAL 4–5	1.02 (0.5–2.3)	0.943
Multiple foci on mpMRI	3.2 (1.8–5.9)	0.000
Base location for the index lesion	2.3 (1.3–4.3)	0.006
Early extraprostatic extension	2.4 (1.3–4.4)	0.006
**Biopsy criteria**		
Percentage of targeted positive cores (per 20% increment)	1.22 (1.0–1.5)	0.036
Percentage of systematic positive cores (per 20% increment)	1.32 (1.0–1.7)	0.027
**Constant**^a^	0.03 (0.01–0.08)	<0.001

CI = confidence interval; mpMRI = multiparametric magnetic resonance imaging; OR = odds ratio; PI-QUAL = Prostate Imaging Quality score (nondiagnostic, PI-QUAL 1–2; sufficient, PI-QUAL 3; optimal, PI-QUAL 4–5).

a Residual from the multivariable analysis.

## 4 Discussion

This multicenter study provides evidence that mpMRI quality, assessed is terms of the PI-QUAL score, is related to clinical outcomes. Nondiagnostic mpMRI quality (PI-QUAL <3) was significantly associated with an increase in upstaging from organ-confined disease on mpMRI to locally advanced disease on radical prostatectomy pathology (pT3a–b), as well as lower detection rates for PI-RADS 5 lesions and EPE and a lower number of suspicious lesions, when compared to PI-QUAL ≥3. No significant differences were observed when the mpMRI quality increased from sufficient (PI-QUAL 3) to optimal (PI-QUAL >3).

The higher proportion of PI-RADS 5 lesions identified in the PI-QUAL 3 (42.1%) and PI-QUAL >3 (48%) groups than in the PI-QUAL<3 group (27.5%) could have important clinical implications. Many clinicians will retain a high degree of suspicion of csPCa when confronted with a PI-RADS 5 lesion, since as many as 83–85% of these patients present with csPCa and usually require active treatment, compared to 52–60% of patients with PI-RADS 4 and 12–20% of those with PI-RADS 3 lesions [2], [13]. A higher rate of detection for PI-RADS 5 lesions could lead to a higher rate of repeat biopsies in cases of negative or clinically insignificant PCa on initial biopsy, while such additional diagnostic procedures may not have been performed in cases with a lower PI-RADS score [14].

Regarding local staging, the mpMRI T stage corresponded exactly to the stage on pathology for the radical prostatectomy specimen in 71.2% of cases. However, pathological upstaging was more frequently observed for nondiagnostic quality mpMRI (35%) than for PI-QUAL ≥3 (19.3%). This implies that despite the added value of preoperative mpMRI and targeted biopsies, staging before surgery was still inaccurate for one in three patients. High-quality mpMRI mitigates this risk, but there is still room for improvement, as one in five patients was still misclassified.

Early EPE had a moderate to low sensitivity and specificity for prediction of locally advanced disease on adequate-quality mpMRI, while it had higher diagnostic value for nondiagnostic images, which could be a sign of underestimation of the local extent. Late EPE, only visible on mpMRI of sufficient or optimal quality, had excellent specificity (95% and 87%, respectively), low sensitivity, and acceptable positive predictive and negative values, although the results are lower than in the literature [11]. An additional point to note is that the incidence of late EPE in the PI-QUAL ≥3 group could be a contributory factor for the higher proportion of PI-RADS 5 lesions in this group.

Another explanation for the higher upstaging rate in the PI-QUAL <3 group may be the greater detection of multiple suspicious lesions identified with increasing mpMRI quality (15% for nondiagnostic vs 33.3% for sufficient vs 35% for optimal mpMRI quality). This is in accordance with the risk of missing significant lesions associated with the definition of a nondiagnostic-quality PI-QUAL score. Our multivariable analysis supports previous findings, since nondiagnostic imaging quality, multiple suspicious lesions, and the presence of early EPE were independently associated with upstaging on pathology. Clinicians should therefore be highly cautious in cases of late EPE on sufficient-quality images, and should also be aware when planning nerve-sparing surgery that nondiagnostic-quality mpMRI may miss or underestimate EPE.

The PI-QUAL score and its clinical importance have been assessed in several other studies. Inter-reader agreement varied from moderate (κ coefficient 0.51) to strong (κ coefficient 0.85) [9], [15], [16], [17]. In one of the studies showing moderate agreement, sufficient quality (PI-QUAL ≥3) was associated with excellent PI-RADS version 2.1 agreement (κ coefficient 0.88), while another study showed lower uncertainty in the diagnostic pathway (less frequent PI-RADS 3 scores and a greater ability to rule in cancer) with increasing PI-QUAL score [16], [17]. Pötsch et al [18] recently reported the use of PI-QUAL in the mpMRI-transrectal ultrasound fusion biopsy pathway and found lower PCa prevalence among patients with PI-QUAL ≤3, with potential for reducing the number of biopsies performed, but they did not show an increase in diagnostic performance with increasing imaging quality. However, the authors performed a pooled analysis of patients with imaging of nondiagnostic or sufficient quality in comparison to patients with optimal imaging. Our findings confirm the benefit of having mpMRI of sufficient quality; however, the added-value of optimal quality, as underlined by Pötsch et al, remains unclear. Finally, whether and when mpMRI should be repeated in cases of nondiagnostic quality is still an open question, since it may impact the surgical strategy, as well as the length of androgen deprivation and the radiation template in cases undergoing nonsurgical treatment. When nondiagnostic quality is found after biopsy (eg, for referred external patients), the optimal duration before repeat mpMRI must be established and weighed in balancing the benefits and risks for cases with aggressive lesions.

### 4.1 Strengths and limitations

Our study has several strengths. First, we report the largest collective mpMRI quality assessment by expert radiologists using the standardized PI-QUAL score. Second, the study provides real-life data and shows the heterogeneity of imaging quality among different European centers. Third, we provide further evidence that assessing mpMRI quality via the PI-QUAL score has a significant impact on adequate disease staging; therefore, local staging based on mpMRI with a PI-QUAL score of <3 should be interpreted with caution, especially when nerve-sparing surgery is planned.

Our study also has some limitations. First, because of the multicenter design of the study and the large number of patients included, there was no central review of all mpMRI examinations by a single radiologist; therefore, no inter-reader agreement could be assessed, and staging or EPE interpretation may have differed among the centers. However, evidence has shown a moderate to high degree of inter-reader agreement regarding PI-QUAL assessment and high inter-reader agreement for PI-RADS, and all the radiologists were experts (>200 reads/yr and >1000 mpMRI reads in total). Second, regarding EPE, since no consensus exists on the best-performing score, we used the score described by Pesapane because of its high AUC. However, other scores with similar performance may be used by other centers, limiting the external validity of our results [11], [19], [20]. Interestingly, a recent study showed that combining some of the imaging features of PI-RADS version 2.1 with an “mEPE-score” provides additional discriminating ability and could help in standardization in the future [8]. Third, the heterogeneity in PI-QUAL distribution among centers, and the fact that one center contributed only optimal-quality mpMRI, may raise questions regarding the mpMRI reading. This only reflects a very rigorous protocol with repeat mpMRI acquisition in cases of suboptimal imaging. Fourth, the distribution of PI-QUAL scores was not even across the centers. Interestingly, 18 of the 37 nondiagnostic-quality mpMRI scans from the center with the highest PI-QUAL 1–2 numbers were for patients referred after undergoing mpMRI at an external center; this underlines the importance of centralizing and reviewing images, especially those performed in low-volume imaging centers. A review of the scoring sheets revealed that the main reason for nondiagnostic imaging was an inadequate dynamic contrast-enhanced sequence in 18/37 examinations (seminal vesicles not covered, inadequate temporal resolution, inadequate slice thickness, presence of gaps, and repetition artifacts). Other frequent causes were an inadequate field of view on T2-weighted or diffusion-weighted imaging, and an inadequate high *b* value. In addition, even if radiologists scored each subitem on the PI-QUAL scoring sheet, only global scores are reported here. Ultimately, the study population consisted only of patients who had a lesion on mpMRI and were treated with prostatectomy. Therefore, the current findings may not be applicable to patients who do not present with a significant lesion on mpMRI, and do not answer whether global improvements in mpMRI quality will have an impact on the global diagnostic pathway for patients suspected of having csPCa.

## 5 Conclusions

We report the first large multicenter study using the PI-QUAL classification and provide further evidence that PI-QUAL <3 is an adequate cutoff for nondiagnostic quality of mpMRI. Nondiagnostic quality on mpMRI has a clinical impact: a greater rate of upstaging from organ-confined PCa on radiology to locally advanced (pT3a–b) disease on pathology, lower detection rates for PI-RADS 5 lesions and extraprostatic extension, and a lower number of suspicious lesions detected.

  ***Author contributions***: Olivier Windisch had full access to all the data in the study and takes responsibility for the integrity of the data and the accuracy of the data analysis.

  *Study concept and design*: Windisch, Benamran, Fiard, Diamand, Oderda.

*Acquisition of data*: Windisch, Benamran, Dariane, Martins Favre, Djouhri, Chevalier, Guillaume, Gatti, Faletti, Colinet, Lefebvre, Bodard.

*Analysis and interpretation of data*: Windisch, Benamran, Dariane, Oderda, Bodard, Diamand, Fiard.

*Drafting of the manuscript*: Windisch, Diamand, Fiard.

*Critical revision of the manuscript for important intellectual content*: Benamran, Dariane, Martins Favre, Djouhri, Oderda, Gatti, Faletti, Colinet, Bodard, Diamand, Fiard.

*Statistical analysis*: Windisch.

*Obtaining funding*: None.

*Administrative, technical, or material support*: None.

*Supervision*: Benamran, Fiard, Oderda, Diamand.

*Other*: None.

  ***Financial disclosures:*** Olivier Windisch certifies that all conflicts of interest, including specific financial interests and relationships and affiliations relevant to the subject matter or materials discussed in the manuscript (eg, employment/affiliation, grants or funding, consultancies, honoraria, stock ownership or options, expert testimony, royalties, or patents filed, received, or pending), are the following: None.

  ***Funding/Support and role of the sponsor*:** None.
