# Supplementary material for: Role of the Prostate Imaging Quality PI-QUAL Score for Prostate Magnetic Resonance Image Quality in Pathological Upstaging After Radical Prostatectomy: A Multicentre European Study
Source: Eur Urol Open Sci. 2022 Dec 15;47:94–101. doi: 10.1016/j.euros.2022.11.013 (PMC9806708; doi:10.1016/j.euros.2022.11.013)
Supplement: Supplementary data 1 [file mmc1.docx]

**Supplementary Table 1 – Radiologist experience by center**

| Centers | Number of radiologist | Number of MRI per year* | Number of MRI read* | Years of experience |
| --- | --- | --- | --- | --- |
|  |  |  |  |  |
| #1 | 1 | >200 | >1000 | 10 years |
| #2 | 2 | >200 | >1000 | 4 and 12 years |
| #3 | 2 | >200 | >1000 | 7 and 15 years |
| #4 | 2 | >200 | >1000 | 3.5 and 7 years |
| #5 | 1 | >200 | >1000 | 10 years |
|  |  |  |  |  |
| *ESUR/ESUI consensus-based criteria for "expert" radiologists = minimum 1000 cases read and minimum 200 cases per year | | | | |

**Supplementary Table 2 - mpMRI sensitivity and specificity of extracapsular extension (ECE) for detection of locally advanced disease on prostatectomy specimen**

| **Agregated ECE **** | Non-diagnostic* (n=40) | Sufficient* (n=57) | Optimal* (n=254) |
| --- | --- | --- | --- |
| Sensitivity | 36% (13-65) | 67% (41-87) | 65% (54-75) |
| Specificity | 85% (65-96) | 72 %(55-85) | 52% (44-60) |
| PPV | 56% (21-86) | 52% (31-73) | 43% (34-51) |
| NPV | 71.0% (52-85) | 82% (66-93) | 73% (62-79) |
| AUC | 0.6 (0.45-0.75) | 0.69 (0.56-0.83) | 0.58 (0.52-0.65) |
| **Early ECE**** |  |  |  |
| Sensitivity | 36% (13-65) | 28% (10-53) | 22% (14-32) |
| Specificity | 85% (65-96) | 77% (61-89) | 66% (58-74) |
| PPV | 56% (21-86) | 36% (13-65) | 27% (17-38) |
| NPV | 71.0% (52-85) | 70% (54-83) | 60% (53-68) |
| AUC | 0.6 (0.45-0.75) | 0.52 (0.40-0.65) | 0.44 (0.39-0.50) |
| **Late ECE **** |  |  |  |
| Sensitivity | NA | 39% (17-64) | 43% (32-54) |
| Specificity | NA | 95% (83-99) | 87% (80-91) |
| PPV | NA | 78% (40-97) | 64% (51-76) |
| NPV | NA | 77% (63-88) | 73% (66-79) |
| AUC | NA | 0.67 (0.55-0.79) | 0.65 (0.59-0.70) |

Locally advanced disease refers to pT3a or pT3b staging on radical prostatectomy specimen. Data are presented as mean (95% confidence interval). *Image quality as assessed with the PI-QUAL score ; non-diagnostic refers to PI-QUAL 1-2, sufficient refers to PI-QUAL 3, and optimal refers to PI-QUAL 4-5. **Extracapsular extension is defined as late or early according to Pesapane’s definition. Aggregated indicated the presence of either early or late extracapsular extension.

Abbreviations: ECE, extracapsular extension; PPV, positive predictive value; NPV, negative predictive value, AUC, area under the curve.

**Supplementary Table 3 – Pre-operative biopsy results**

|  | Non-diagnostic* (n=40) | Sufficient*(n=57) | Optimal* (n=254) | **P value** |
| --- | --- | --- | --- | --- |
| **Biopsies** |  |  |  |  |
| Median number of targeted cores | 4 (3-5.5) | 4 (3-5) | 4 (4-6) | 0.328 |
| Median number of positive targeted cores | 3 (2.5-4) | 3 (2-4) | 3 (1-4) | 0.654 |
| Mean number of systematic cores | 9 (8-11) | 10 (8-12) | 11 (8-12) | 0.123 |
| Median number of positive systematic cores | 2 (1-4) | 2 (1-3) | 2 (1-4) | 0.048 |
| csPCa in targeted biopsy | 27 (67.5) | 37 (64.9) | 180 (70.9) | 0.649 |
| csPCa in systematic biopsy | 22 (55.0) | 29 (50.9) | 149 (58.7) | 0.543 |
| Upgrading on targeted biopsy compared to systematic | 9 (22.5) | 18 (31.6) | 92 (36.2) | 0.216 |
| Biopsy highest ISUP score |  |  |  | 0.628 |
| ISUP 1 | 11 (27.5) | 15 (26.3) | 49 (19.3) |  |
| ISUP2 | 15 (37.5) | 18 (31.5) | 105 (41.3) |  |
| ISUP3 | 5 (12.5) | 12 (21.1) | 59 (23.2) |  |
| ISUP4 | 7 (17.5) | 9 (15.8) | 32 (12.6) |  |
| ISUP5 | 2 (5) | 3 (5.3) | 9 (3.6) |  |

*Image quality as assessed with the PI-QUAL score ; non-diagnostic refers to PI-QUAL 1-2, sufficient refers to PI-QUAL 3, and optimal refers to PI-QUAL 4-5.

**Supplementary Table 4 – Univariate logistic regression values for preoperative variables associated with pathological upstaging in patients with mpMRI-confined disease**

| **Risk of pathological upstaging to locally invasive disease with organ-confined mpMRI T-stage** | Odds ratio | 95% CI | P value |
| --- | --- | --- | --- |
| **mpMRI criteria** |  |  |  |
| **PI-QUAL score (control = PI-QUAL 3)** |  |  | **0.067** |
| - PI-QUAL <3 | **2.53** | 0.98-6.5 | **0.053** |
| - PI-QUAL>3 | 1.12 | 0.53-2.4 | 0.761 |
| PI-RADS (control = PI-RADS 3)* |  |  | 0.315 |
| - PI-RADS 4 | 1.34 | 0.37-4.82 | 0.656 |
| - PI-RADS 5 | 1.91 | 0.53-6.86 | 0.318 |
| ROI Maximum diameter (mm) | 1.03 | 0.99-1.08 | 0.111 |
| **More than one suspicious lesions** | **2.15** | 1.27-3.65 | **0.004** |
| Location of ROI |  |  |  |
| - Apex | 0.61 | 0.33-1.11 | 0.107 |
| - Mid | 1.26 | 0.73-2.16 | 0.409 |
| **- Base** | **1.68** | 0.97-2.89 | **0.063** |
| - Posterior | 1.23 | 0.65-2.36 | 0.523 |
| - Anterior | 0.98 | 0.53-1.79 | 0.935 |
| - Peripherical | 1.50 | 0.60-3.73 | 0.380 |
| **- Transitional** | **0.32** | 0.09-1.07 | **0.064** |
| Tumor volume (by ml) | 1.06 | 0.94-1.19 | 0.350 |
| Prostate volume (by ml) | 1.00 | 0.99-1.01 | 0.907 |
| **Demographic and clinical criteria** |  |  |  |
| **PSA (ug/L)** | **1.04** | 1.00-1.07 | **0.036** |
| PSA density (ug/L/ml) | 1.56 | 0.74-3.33 | 0.241 |
| **Age (by increment of 10 years)** | **1.54** | 1.03-2.33 | **0.037** |
| Clinical stage (control cT1) |  |  | **0.459** |
| - cT2 | 1.31 | 0.77-2.23 | 0.309 |
| - cT3 | 0.64 | 0.14-2.95 | 0.566 |
| **Biopsy results** |  |  |  |
| Global highest ISUP (control = ISUP1) |  |  | 0.349 |
| - ISUP2 | 1.41 | 0.66-3.04 | 0.374 |
| - ISUP3 | 1.81 | 0.78-4.14 | 0.163 |
| - ISUP4 | 1.94 | 0.78-4.84 | 0.156 |
| - ISUP 5 | **3.23** | 0.91-11.5 | **0.069** |
| Percentage of targeted cores with csPCa (by increment of 20%)** | **1.23** | 1.04-1.45 | **0.014** |
| Percentage of systematic cores with csPCa (by increment of 20%)** | **1.45** | 1.17-1.81 | **0.001** |

*PI-RADS <3 dropped because predicts outcomes perfectly

** is presented by increment of 20% and not continuously for better understanding and readability.

Abbreviations ; ROI – region of interest (defined as the target with the highest PI-RADS score, or the largest target in case of multiple targets with equivalent PI-RADS score)
